# REPEAT AND SERIAL EMERGENCY DEPARTMENT (ED) OLDER ADULT USERS—A STATE-LEVEL ANALYSIS USING THE NYU-JHU ALGORITHM

**DOI:** 10.1093/geroni/igad104.2336

**Published:** 2023-12-21

**Authors:** Uma Kelekar, Debasree Das Gupta, Amel Ben Abdesslem, Diep Tran, Jewel Shepherd, Sidney Turner

**Affiliations:** Marymount University, Arlington, Virginia, United States; Utah State University, Logan, Utah, United States; Marymount University, Arglington, Virginia, United States; Johns Hopkins University School of Medicine, Baltimore, Maryland, United States; University of South Dakota, Vermillion, South Dakota, United States; Fors Marsh Group LLC, Falls Church, Virginia, United States

## Abstract

Older adults account for 12-24% of all Emergency Department (ED) visits and studies have shown a 25-35% increase in older adult ED visits over time. While some studies have analyzed ED encounters over a single healthcare system, a year or a specific condition, research comparing the long-term frequent use of the ED across cohorts of older adults in the United States is limited. We address this gap by identifying characteristics associated with repeat (>1 visit during the time period) and serial/frequent (>=4/year) ED utilization for Maryland. Using the 2017-2019 State Emergency Department Databases (SEDD), we extracted ED visits among the elderly (>=60 years) population. By applying the modified New York University Algorithm by John Hopkins University (NYU-JHU-EDA), we classified ED visits into 11 categories, and we conducted bivariate and multivariate analyses to identify inter-age variations in the type of visits to the ED across single, repeat and serial users, after controlling for covariates. Of the total 5,331,843 ED visits between 2017 and 2019, 1,244,878 (~23%) were older adult visits. Relative to the 60–69-year-olds, those 80 and above had higher odds of being repeat users for emergent issues [OR=1.34, CI:1.07-1.67], primary-care treatable condition [OR=1.29, CI:1.03-1.62], and severe [1.30, CI: 1.03-1.64] and non-severe injuries [1.61, CI:1.28-2.01]. Relative to 60-69 year olds non-serial users, the oldest cohort demonstrated lower odds for psychiatric/alcohol and drug-related diagnoses [OR=0.63, CI:0.42, 0.95]. Our findings highlight the need to design age-group specific interventions to reduce the frequent ED utilization among the elderly in Maryland.

